# Predicted body weight relationships for protective ventilation – unisex proposals from pre-term through to adult

**DOI:** 10.1186/s12890-017-0427-1

**Published:** 2017-05-23

**Authors:** Dion C. Martin, Glenn N. Richards

**Affiliations:** ResMed Science Center, ResMed Ltd, 1 Elizabeth Macarthur Drive, Bella Vista, 2153 Sydney, Australia

**Keywords:** Pediatrics, Tidal volume, Ventilator-induced lung injury, Body weight, Height, Algorithms, Ideal body weight, Mechanical ventilators, Growth charts, Adult

## Abstract

**Background:**

The lung-protective ventilation bundle has been shown to reduce mortality in adult acute respiratory distress syndrome (ARDS). This concept has expanded to other areas of acute adult ventilation and is recommended for pediatric ventilation. A component of lung-protective ventilation relies on a prediction of lean body weight from height. The predicted body weight (PBW) relationship employed in the ARDS Network trial is considered valid only for adults, with a dedicated formula required for each sex. No agreed PBW formula applies to smaller body sizes. This analysis investigated whether it might be practical to derive a unisex PBW formula spanning all body sizes, while retaining relevance to established adult protective ventilation practice.

**Methods:**

Historic population-based growth charts were adopted as a reference for lean body weight, from pre-term infant through to adult median weight. The traditional ARDSNet PBW formulae acted as the reference for prevailing protective ventilation practice. Error limits for derived PBW models were relative to these references.

**Results:**

The ARDSNet PBW formulae typically predict weights heavier than the population median, therefore no single relationship could satisfy both references. Four alternate piecewise-linear lean body-weight predictive formulae were presented for consideration, each with different balance between the objectives.

**Conclusions:**

The ‘PBWuf + MBW’ model is proposed as an appropriate compromise between prevailing practice and simplification, while also better representing lean adult body-weight. This model applies the ARDSNet ‘female’ formula to both adult sexes, while providing a tight fit to median body weight at smaller statures down to pre-term. The ‘PBWmf + MBW’ model retains consistency with current practice over the adult range, while adding prediction for small statures.

**Electronic supplementary material:**

The online version of this article (doi:10.1186/s12890-017-0427-1) contains supplementary material, which is available to authorized users.

## Background

Historically, mechanical ventilation was initiated with a tidal volume based on a patient’s actual body weight, which was believed to reflect metabolic need. This remains common practice for pediatric ventilation, and for much of adult ventilation. However, a ‘lung-protective strategy’ is increasingly the standard of care for acute ventilation based on data showing that this approach to treating acute respiratory distress syndrome (ARDS) in adults was associated with reduced mortality [1–3]. Lung-protective ventilation has also been shown to improve outcomes in patients ventilated in the operating room and in the intensive care unit (ICU) [4, 5]. Recent consensus guidance recommends the lung-protective strategy also be applied in pediatric acute lung injury [6].

Key elements of a lung-protective strategy are the application of positive end-expiratory pressure (PEEP), limitation of plateau pressure, and a minimal tidal volume scaled to a ‘predicted’, rather than actual, body weight. The use of predicted weight is based on the assumption that volutrauma might be minimized by delivering a volume appropriate to the patient’s lung capacity [1]. Lung capacity and respiratory system compliance relate more closely to height than to weight, at least in normal subjects. Therefore, by calculating initial tidal volume based on predicted (or lean) body weight rather than actual weight, configuration of the ventilator retains some connection to metabolic need (weight), while also reducing potential for volutrauma (height). The tidal volume scaling factor is 5–8 mL/kg of predicted body weight (PBW) (or less at elevated plateau pressure) [1, 6].

Despite consensus in favor of lung protective ventilation, multiple surveys suggest that adherence is not uniform, with much scope for improvement [7–9]. Various initiatives have been suggested to improve adherence, such as to change routine charting practices from the absolute tidal volumes (mL) to mL/kg_PBW_ [8, 10], or to configure alarms around mL/kg_PBW_ rather than absolute volumes [11]. Such initiatives may be hindered by the multiple challenges in predicting weight from height. There is the challenge of obtaining a reasonable estimation of height (an issue not limited to protective ventilation alone). If height cannot be measured or provided by the patient, more convenient surrogate measures such as arm-span, arm demispan [12], ulna length, or knee height have been devised. There is the challenge of appropriately estimating a weight from the estimated height. For adults, PBW formulae do exist, with 2 different approaches used to predict lean body mass in the early ARDS studies [1, 13]. The difference in estimated PBW between these 2 approaches can vary by up to 30% [14, 15]. Consequently standardization has been proposed [15]. Meanwhile, for pediatric ventilation, there is no simple formula to estimate PBW: the dominant PBW formula (used in the ARDS Network trial and generally attributed to Devine [16]) is formally defined only for heights above about 5 ft/152 cm. Recent publications have emphasized the challenge and complexity of extending protective ventilation into pediatrics [6, 17, 18]. For instance, growth charts may be required to estimate an ideal/predicted body weight from an estimated height/length (or surrogate). Finally, the most established PBW formula [1, 19] includes the patient’s sex in addition to height, but it is unclear whether this is justified, particularly given the inaccuracies elsewhere in the process. A unisex formula may simplify the task of applying lung-protective ventilation, and simplification may reduce error rates [20].

This analysis determined whether it was feasible for body-weight predictive formulae to be unisex and to span all body sizes (from infants to adults), while retaining adequate relevance to the outcomes of the landmark ARDSNet protective ventilation study.

## Methods

Relevant clinical and biomedical engineering literature were identified by searching PubMed (up to June 2016). Search terms were: protective ventilation, predicted body weight, ideal body weight, pediatric ventilation, growth charts, and related terms. Publications were reviewed primarily from the viewpoint of protective ventilation, rather than pharmacokinetics or other applications of weight estimation.

**Fig. 1:**
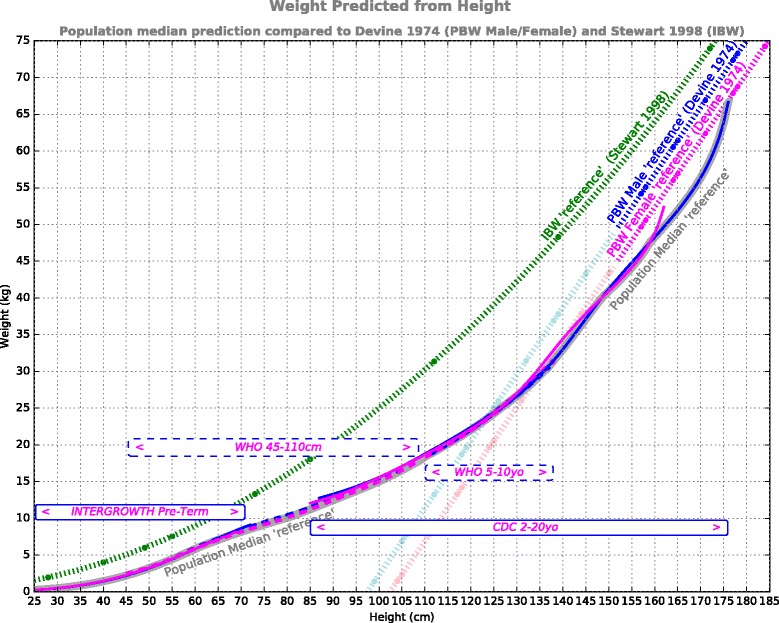
Weight from Height. Shown in *blue* (male) and *magenta* (female) are median population height-weight data from 4 data sources: Pre-term infants [24] (*INTERGROWTH Pre-Term*), birth to 2 years WHO data [21] (*WHO 45–110 cm*, dashed), 5 to 10 years WHO data [21] (*WHO 5-10yo*), and 2 to 20 year CDC data [22] (*CDC 2-20yo*). The Population Median ‘reference’ derived from these data is shown in thick grey. The PBW Male/Female formulae used in the 2000 ARDS Network trial [19] is shown dotted (*blue/magenta*). The IBW reference used in the 1998 Stewart trial [13] is shown (*dotted*) in *green*

An ideal data set for this feasibility analysis would have been known lean body weights for all statures across a broad population. The literature search did not divulge this ‘ideal’ data set. Instead, ‘lean’ body weight was assumed to be adequately represented by median weight during growth across healthy populations. One dataset allowed median weight to be *directly* related to height over the 45–110 cm range, the weight-for-recumbent length standard compiled by the World Health Organization (WHO) [21]. This is a recommended resource for monitoring growth across a nominally 0–2 year age [21, 22], and represents approximately 8500 ethnically and culturally diverse children, from birth to 5 years, under favorable environmental conditions. For ages/heights either side of this range, direct weight-stature population data were not found. For larger body sizes, 2 age-based data sets were applied. The US Centers for Disease Control (CDC) publish weight-for-age & height-for-age growth data over the 2- to 20-year-old span, drawn from multiple US national health surveys, with a minimum requirement of 400 subjects represented at each age point. Similarly the WHO publishes weight-for-age & height-for-age growth data for the 5- to 10-year-old span [21]. In a method similar that of McLaren [23], weight-for-stature relationships were synthesized from these age-based data: within a given population, 50^th^ centile age-weight data were age-mapped to 50^th^ centile age-height data to infer weight-from-height. The same approach was applied to synthesize weight-for-height for the smallest body sizes (25 to 72 cm), using postmenstrual age-based data from the Preterm Postnatal follow-up to the INTERGROWTH-21^st^ Project [24]. This study drew from 201 subjects (99 boys, 102 girls) at 8 global locations. The resulting weight-for-stature composite of these 4 data sources are plotted in Fig. 1, labelled *INTERGROWTH Pre-Term*, *WHO 45–110 cm*, *WHO 5-10yo*, and *CDC 2-20yo*. The data span from 25 cm body length up to the median adult male height (177 cm). Given the protective ventilation emphasis on lean body weight, these 4 data sets were processed to extract a single curve representing the lowest weight associated with each height within these data (Fig. 1, thick grey curve labelled *Population Median ‘reference’*). This curve thereafter acted as a unisex surrogate for lean body weight when developing the PBW models.

Superimposed in Fig. 1 are the 2 most common relationships for predicting body weight used in protective ventilation, the Devine formulae adopted for the ARDS Network study of 2000 [1, 19], labelled *PBW Male ‘reference’* and *PBW Female ‘reference’*, and the relationship used by Stewart et al. (1998) [13], labelled *IBW ‘reference’*.

The key measure of ‘fit’ used for each PBW model was error relative to the population median reference, expressed as a percentage. Relative error was used to ensure that tightness of fit was scaled with height, in contrast to absolute error used in traditional linear regression. Error beyond 176 cm height was not able to be assessed because this was where median height plateaued in the CDC data.

For each model of interest, a piecewise linear curve was fitted, with the objective of using the fewest segments capable of ensuring <5% over-estimation of weight across the height/length range when compared with the population median reference. An underestimation tolerance of up to 10% was allowed based on the premise that if trade-off was needed, under-estimation is preferred to over-estimation due to the concerns of excessive volume in lung-protective ventilation. There was also a specific consideration that any formula aimed at lung-protective ventilation must retain relevance to clinically proven best practice. Therefore, over heights where the established male/female PBW formulae might be considered valid, they provided alternate ‘references’ with similar error margins (that is, the permissible error target was set to be no more than 5% above the PBW male/female references [19], but if necessary up to 10% below these references).

**Fig. 2:**
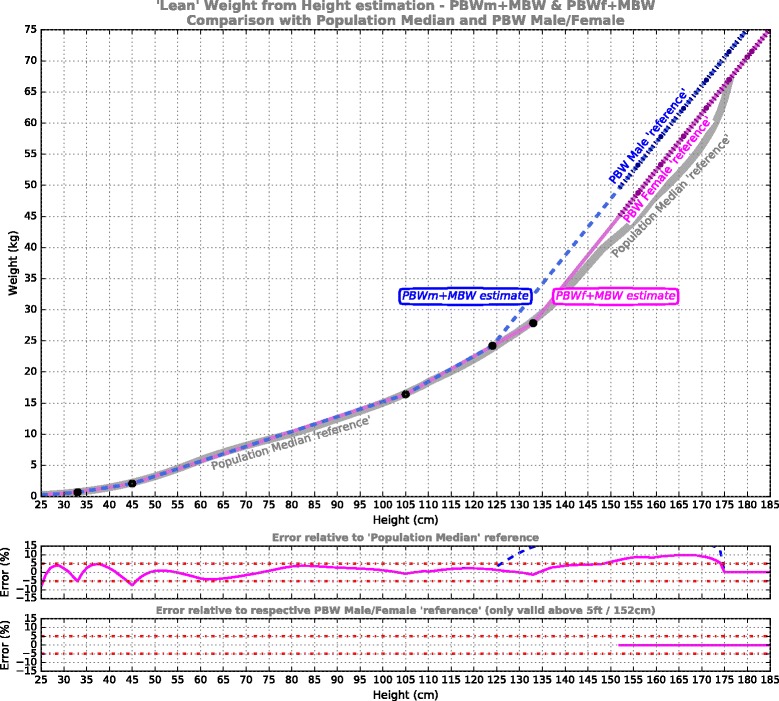
‘Lean’ weight from height estimation for the models PBWm + MBW (*dashed blue*) and PBWf + MBW (*magenta*). These adhere to the Devine PBW references [19] (shown dotted) where viable, but at smaller statures adopt a unisex extension targeting the Population Median reference (shown in grey). Shown underneath is the error performance for the model relative to the respective reference curves (male – *blue*, female – *magenta*)
- **PBWu + MBW** model: This single curve plots a unisex (PBWu) mid-path between the established male/female PBW formulae, with MBW extensions for small body-sizes (Fig. 3).

Applying these fitting strategies resulted in the following PBW models:

- **PBWmf + MBW** model: These 2 curves fully adhere to the established adult male & female PBW reference formulae (PBWm/PBWf), so represent minimum change from current ventilation practice, but add a unisex extension for small body-sizes based on median body weight (MBW), charted in Fig. 2.

**Fig. 3:**
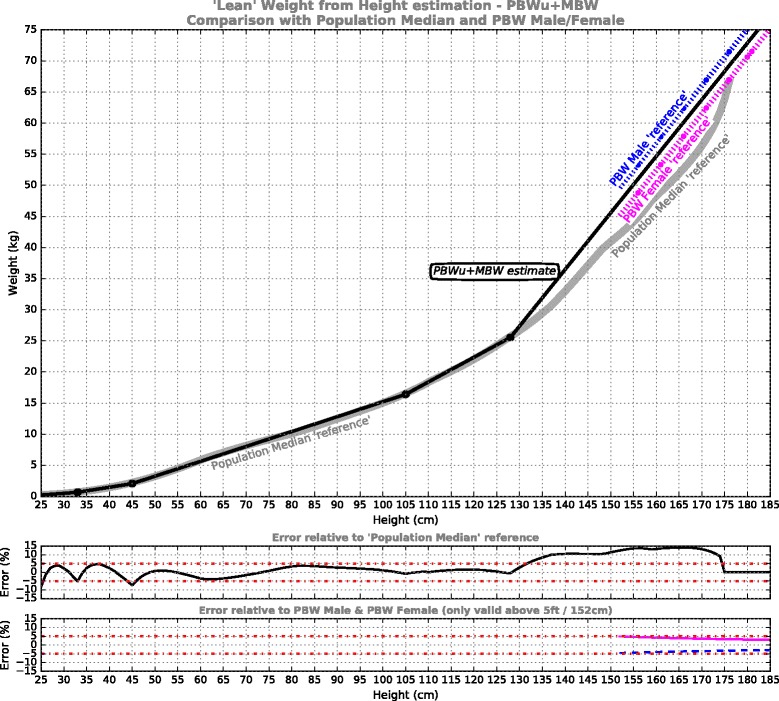
‘Lean’ weight from height estimation for the PBWu + MBW model, comprising a unisex mid-path between the established male/female PBW formulae [19] at adult statures, while targeting the Population Median reference at smaller statures. Shown underneath is error performance for the model relative to the reference curves
- **PBWuf + MBW** model: This curve re-purposes the established female formula as a unisex formula for larger body sizes (PBWuf), given that – as is evident from Fig. 1 – the established PBW female formula is the closest approximation to ‘lean body weight’ amongst the 3 traditional formulae. MBW extensions are added for small body-sizes (Fig. 4).

**Fig. 4:**
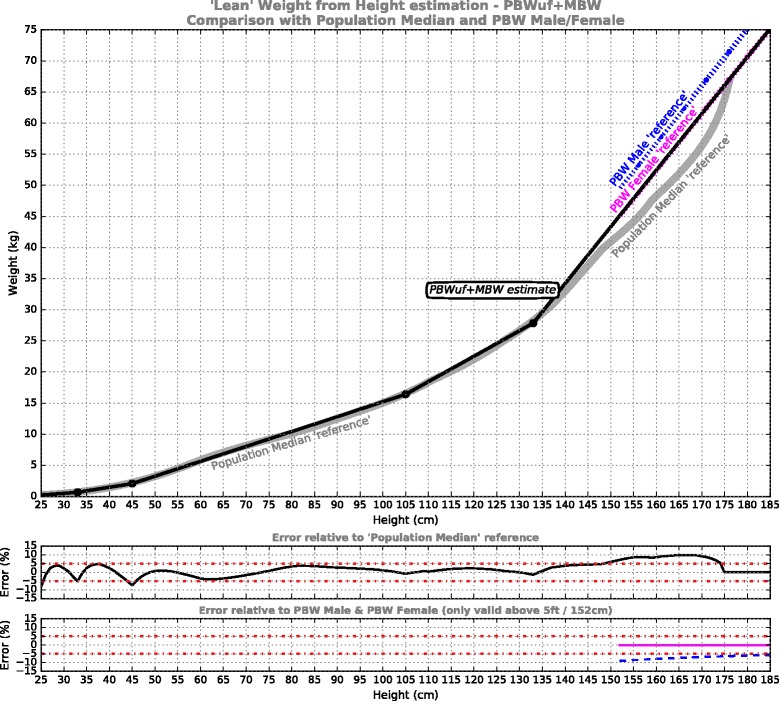
‘Lean’ weight from height estimation for the PBWuf + MBW model, which adopts the Devine PBW Female formula [19] as the unisex model at adult statures, then targets the Population Median reference at smaller statures. Shown underneath is error performance for the model relative to the reference curves
- **MBW** model: This unisex curve targets minimal error relative to the population median reference across the entire height range, i.e. abandons attachment to the established formulae (Fig. 5).

**Fig. 5:**
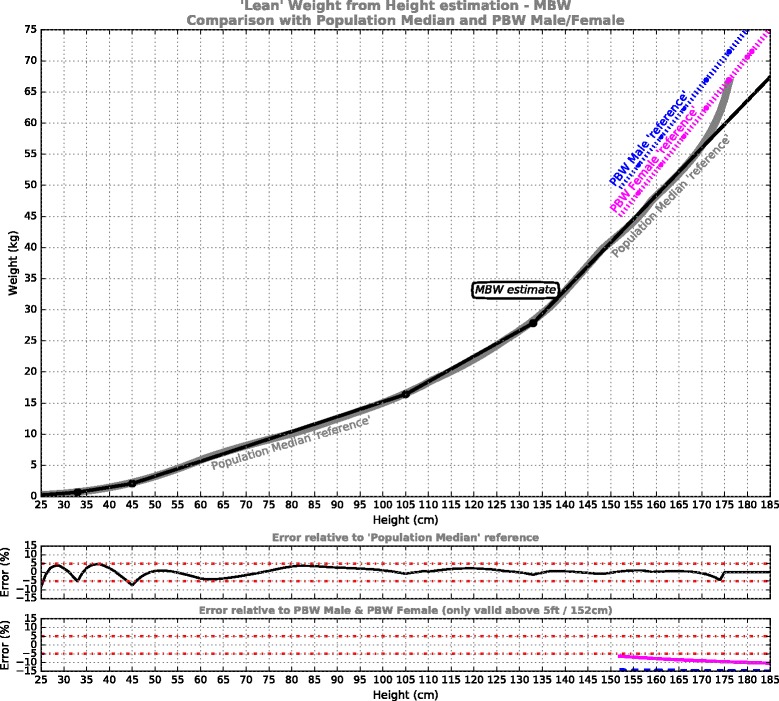
‘Lean’ weight from height estimation for the MBW curve, targeting the Population Median reference (in grey) across the entire height range. This model departs entirely from the PBW Male/Female ‘references’ [19], shown (dotted) for comparison. Shown underneath is error performance for the model relative to the reference curves

Calculations and interpolation, were implemented in MathCad 15 (2011 Parametric Technology Corporation). Graphing was performed within the iPython environment [25] via Anaconda Software Distribution [26].

A data supplement is provided (see Additional file 1), including the derived population median ‘reference’ curve, the 4 weight-height population data sets, calculators for the above 4 models, and the established PBW Male/Female calculation for comparison. Also included are imperial conversions for the formulae constants and coefficients within each piece-wise linear model.

## Results

Figure 1 demonstrates that the established weight predictors (PBW Male, PBW Female, and IBW ‘references’) deviate substantially from the Population Median ‘reference’. Compared to the population median data, the IBW reference grossly over-estimates body weight throughout the height range. The PBW Male/Female formulae are generally recommended only for heights above 5 ft/152 cm [15, 16]. When compared to Population Median reference, these formulae align well at average adult height and in the 125–140 cm range, but elsewhere generally over-estimate lean body weight, with the male formula showing greatest disparity. Below 124 cm the PBW Male/Female formulae become increasingly invalid.

The results of the piecewise linear PBW models are presented in Figs. 2, 3, 4 and 5. For each of the models, five segments were required to satisfy the imposed error target. The first 3 segments are common to all models, from pre-term infants to 105 cm, while above 105 cm the models deviate. The model formulae are presented in Tables 1, 2, 3 and 4 to facilitate calculation. Table 1 defines the PBWmf + MBW relationships, including a dedicated segment for male and female adults, charted in Fig. 2; Table 2 defines the PBWu + MBW curve, charted in Fig. 3; Table 3 defines the PBWuf + MBW curve, charted in Fig. 4; Table 4 defines the MBW curve, charted in Fig. 5.

**Table 1:** PBWmf + MBW models (adhere to PBW Male/Female formulae [19])

Segment #	Height/Length	Relationship	Reference data	Error % relative to reference
1	Centimeters: 25-33	*PBW* = 0.22 + 0.055 ⋅ (*length* − 25)	Median data: INTERGROWTH Pre-Term [24]	−8.3 < ϵ < +3.7
		*length* in cm, weight in kg		
2	Centimeters: 33*–*45	*PBW* = 0.66 + 0.116 ⋅ (*length* − 33)	Median data: INTERGROWTH Pre-Term [24]	−7.6 < ϵ < +4.5
		*length* in cm, weight in kg		
3	Centimeters: 45*–*105	*PBW* = 2.05 + 0.239 ⋅ (*height* − 45)	Median data: INTERGROWTH Pre-Term [24], WHO 45–110 cm [21]	−7.6 < ϵ < +4
		*height* in cm, weight in kg		
		*Male*		
4 M	Centimeters: 105*–*124	*PBW* = 16.4 + 0.41 ⋅ (*height* − 105)	Median data: WHO 45*–*110 cm & 5-10yo [21], CDC 2-20yo [22]	−0.9 < ϵ < +2.4
		*height* in cm, weight in kg		
5 M	Centimeters: ≥124	*PBW* = 24.2 + 0.91 ⋅ (*height* − 124)	PBW Male [19]	0 < ϵ < 0.2 (cf Population Median: 1.7 < ϵ < +19)
		*height* in cm, weight in kg		
		*Female*		
4 F	Centimeters: 105*–*133	*PBW* = 16.4 + 0.41 ⋅ (*height* − 105)	Median data: WHO 45*–*110 cm & 5-10yo [21], CDC 2-20yo [22]	−1.3 < ϵ < +2.4
		*height* in cm, weight in kg		
5 F	Centimeters: ≥ 133	*PBW* = 27.9 + 0.91 ⋅ (*height* − 133)	PBW Female [19]	0.1 < ϵ < 0.2 (cf Population Median: −1.3 < ϵ < +10)
		*height* in cm, weight in kg		

*ARDS* acute respiratory distress syndrome, *CDC* Centers for Disease Control, *cf* compared with, *kg* kilograms, *PBW* predicted body weight, *WHO* World Health Organization

**Table 2:** PBWu + MBW model (unified curve, compromise between PBW Male and PBW Female formulae [19])

Segment #	Height/Length	Relationship	Reference data	Error % relative to reference
1	Centimeters: 25*–*33	*PBW* = 0.22 + 0.055 ⋅ (*length* − 25)	Median data: INTERGROWTH Pre-Term [24]	−8.3 < ϵ < +3.7
		*length* in cm, weight in kg		
2	Centimeters: 33*–*45	*PBW* = 0.66 + 0.116 ⋅ (*length* − 33)	Median data: INTERGROWTH Pre-Term [24]	−7.6 < ϵ < +4.5
		*length* in cm, weight in kg		
3	Centimeters: 45*–*105	*PBW* = 2.05 + 0.24 ⋅ (*height* − 45)	Median data: INTERGROWTH Pre-Term [24], WHO 45*–*110 cm [21]	−7.6 < ϵ < +4
		*height* in cm, weight in kg		
4	Centimeters: 105*–*128	*PBW* = 16.4 + 0.4 ⋅ (*height* − 105)	Median data: WHO 45*–*110 cm & 5-10yo [21], CDC 2-20yo [22]	−0.9 < ϵ < +1.7
		*height* in cm, weight in kg		
5	Centimeters: ≥ 128	*PBW* = 25.5 + 0.91 ⋅ (*height* − 128)	PBW Male & Female [19]	cf PBW Male: −4.6 < ϵ < −3.2
		*height* in cm, weight in kg		
				cf PBW Female: +3.3 < ϵ < +4.9
				(cf Population Median: −0.8 < ϵ < +14)

*ARDS* acute respiratory distress syndrome, *CDC* Centers for Disease Control, *cf* compared with, *kg* kilograms, *PBW* predicted body weight, *WHO* World Health Organization

**Table 3:** PBWuf + MBW model (unified curve, adopts PBW Female formula for adults [19])

Segment #	Height/Length	Relationship	Reference data	Error % relative to reference
1	Centimeters: 25*–*33	*PBW* = 0.22 + 0.055 ⋅ (*length* − 25)	Median data: INTERGROWTH Pre-Term [24]	−8.3 < ϵ < +3.7
		*length* in cm, weight in kg		
2	Centimeters: 33*–*45	*PBW* = 0.66 + 0.116 ⋅ (*length* − 33)	Median data: INTERGROWTH Pre-Term [24]	−7.6 < ϵ < +4.5
		*length* in cm, weight in kg		
3	Centimeters: 45*–*105	*PBW* = 2.05 + 0.24 ⋅ (*height* − 45)	Median data: INTERGROWTH Pre-Term [24], WHO 45*–*110 cm [21]	−7.6 < ϵ < +4
		*height* in cm, weight in kg		
4	Centimeters: 105*–*133	*PBW* = 16.4 + 0.41 ⋅ (*height* − 105)	Median data: WHO 45*–*110 cm & 5-10yo [21] CDC 2-20yo [22]	−1.6 < ϵ < +2.4
		*height* in cm, weight in kg		
5	Centimeters: ≥ 133	*PBW* = 27.8 + 0.91 ⋅ (*height* − 133)	PBW Female [19]	cf PBW Male: −9.2 < ϵ < −3.8
		*height* in cm, weight in kg		cf PBW Female: −0.1 < ϵ < 0
				(cf CDC 2-20yo: −1.6 < ϵ < +9.8)

*ARDS* acute respiratory distress syndrome, *CDC* Centers for Disease Control, *cf* compared with, *kg* kilograms, *PBW* predicted body weight, *WHO* World Health Organization

**Table 4:** MBW model (unified curve, no adherence to PBW Male/Female formulae [19])

Segment #	Height/Length	Relationship	Reference data	Error % relative to reference
1	Centimeters: 25–33	*PBW* = 0.22 + 0.055 ⋅ (*length* − 25)	Median data: INTERGROWTH Pre-Term [24]	−8.3 < ϵ < +3.7
		*length* in cm, weight in kg		
2	Centimeters: 33-45	*PBW* = 0.66 + 0.116 ⋅ (*length* − 33)	Median data: INTERGROWTH Pre-Term [24]	−7.6 < ϵ < +4.5
		*length* in cm, weight in kg		
3	Centimeters: 45–105	*PBW* = 2.05 + 0.24 ⋅ (*height* − 45)	Median data: INTERGROWTH Pre-Term [24], WHO 45–110 cm [21]	−7.6 < ϵ < +4
		*height* in cm, weight in kg		
4	Centimeters: 105–133	*PBW* = 16.4 + 0.41 ⋅ (*height* − 105)	Median data: WHO 45–110 cm & 5-10yo [21], CDC 2-20yo [22]	−1.6 < ϵ < +2.4
		*height* in cm, weight in kg		
5	Centimeters: ≥ 133	*PBW* = 27.8 + 0.76 ⋅ (*height* − 133)	Median data: WHO 5-10yo [21], CDC 2-20yo [22]	−9.2 < ϵ < +0.9
		*height* in cm, weight in kg		

*CDC* Centers for Disease Control, *kg* kilograms, *PBW* predicted body weight, *WHO* World Health Organization

## Discussion

The first observation from this analysis is that the traditional IBW/PBW formulae of Devine [19] and Stewart [13] are not closely aligned with median body weight across a broad young population, as represented by the 4 data sources [21, 22, 24] (Fig. 1). In addition, as has been reported previously [15], the Devine and Stewart curves are highly dissimilar to each other: the Stewart relationship over-estimates body weight by up to 30% compared with the Devine prediction. The Devine prediction appears to be the most widely used in current practice, and which is associated with the ARDSNet improved mortality outcomes [1].

The rationale behind using PBW in lung-protective ventilation rather than actual body weight might be summarized as:

1. a tidal volume based on actual body weight might lead to volutrauma for an obese patient, or inadequate ventilation in an underweight patient. Within healthy subjects, respiratory system capacity is (non-linearly) related to height [27–29], so height may offer a better basis for initial tidal volume setting.
2. lean body weight is where the vast majority of the body’s metabolic processes occur [16], and is therefore may be better correlated with metabolic requirements than actual weight.
3. hypercapnia might be permissible in favor of avoiding volutrauma.

Thus, when configuring a safe and adequate tidal volume, use of ‘predicted’ or ‘lean’ body weight may better reflect both metabolic requirements and lung size than actual body weight [15]. Even though critical illness and lung injury will likely weaken height-based associations with ventilatory demand and respiratory system characteristics, this approach has proven valuable in improving outcomes [1, 7, 8] when combined with the other elements of lung-protective ventilation.

In this context, the Devine formulae have been applied as a coarse but convenient surrogate for lean body weight [16]. However, as can be seen from Fig. 1, the Devine predictions appear to be not at all ‘lean’ when compared to population median weight over the relevant height range. This disparity may be due to the populations represented by each curve. The Devine prediction is considered to offer reasonable approximation to the body weights of mature adults (aged >18 years) who achieved good longevity [16, 30]. In contrast, the population median represents healthy subjects during growth up to the age of 20 years, surveyed between 1963 and 1994 in the CDC surveys [22]. It seems likely that the growing children and young adults in the population data might not have ‘filled-out’ as much as the healthy older adults of similar height. It is not known if the younger reference population had less muscle or less fat or lighter bones than the Devine population, but the distinction may not be important from the point of view of ventilatory demand. Recent magnetic resonance imaging (MRI) research in adults has revealed that resting energy expenditure is not uniform through all fat-free (lean) mass, but is dominated by highly metabolically active organs compared with bone mass or skeletal muscle [31]. If it is reasonable to assume that organ mass of a growing individual is similar to a mature adult of the same height, the (leaner) population median data may offer a better weight-from-height prediction for the purposes of predicting ventilatory demand than that of a mature adult of similar height. Also of relevance is that as lean body mass increases with height, the metabolically dominant proportion of that mass (organs) does not increase in direct proportion [31]. Hence resting metabolic demand may not be linearly proportional to lean body mass (height) predictions. If so, the use of lean body mass to infer resting ventilatory demand may have some inherent inaccuracy, which will be emphasized in models with steeper mass-per-height gradient, such as the Devine predictions.

**Fig. 6:**
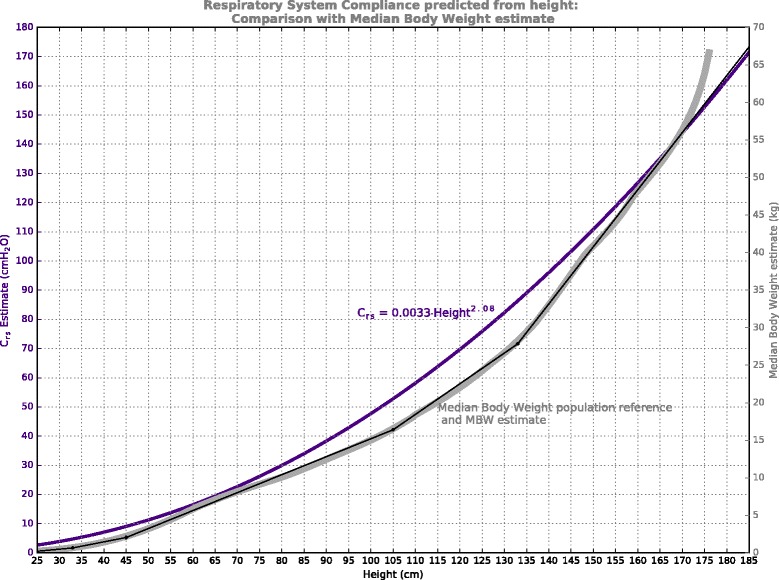
Shape comparison between respiratory system compliance predictions (coloured curve, left axis) and Population Median ‘reference’ and MBW model weight estimates (monochrome curves & right axis). The vertical axes have been adjusted to allow comparison of curve shapes across the height range. The compliance predictions assume normal lung function, supine, and sedated [32]

Even if lean body mass was adequately correlated with ventilatory demand, in pediatric patients it remains to be clinically assessed whether using lean body mass for setting tidal volume might minimize volutrauma. Respiratory system compliance (C_rs_) may be correlated with height [28, 29, 32], but this relationship is highly non-linear. It may be speculated that if the non-linear relationship between lean body weight and height was of similar nature to the non-linear relationship between respiratory compliance and height, then scaling tidal volumes to lean body mass in pediatric ventilation may indeed minimize volume trauma throughout the height range. Such a comparison is presented in Fig. 6, using predictive formulae for normal respiratory system compliance [32] compared to population median body weight. Coarse correlation is evident throughout the height range, with enough similarity to suggest crude proportionality between lean body weight and C_rs_. If so, within normal lungs, the concept of scaling tidal volume to lean body weight may be sympathetic not only to ventilatory demand, but also to appropriate driving pressures. However it must be emphasized that the height-based associations discussed here derive from healthy subjects, not critically ill individuals with injured lungs. Clinical evaluation is needed to determine whether a tidal volume scaled to lean body weight predicted from height can improve mortality while also delivering adequate ventilation.

It is also accepted that any theoretical ‘improvement’ to PBW models may be irrelevant to adult ventilation if the clinical practice built around the ARDSNet findings must be strictly adhered to (i.e. that of basing initial tidal volumes on the Devine body weight predictions, multiplied by 5–8 mL/kg). Even so, for statures outside the ‘proven’ region of the Devine formulae (below 5 ft/152 cm), there may be scope for weight predictions based on population median data.

The curve fitting employed here minimizes relative error (expressed as a proportion of the reference body weight), rather than absolute error as typical in traditional least-squares regression curve fitting. The intent is that the weight predictions maintain fidelity even at the smallest body sizes. As a result, these PBW models may be considered if applying the lung-protective ventilation strategy to children. Across all models, a common piecewise curve is employed up to a height of 105 cm. The PBW models diverge at larger body sizes, reflecting the contextual nature of what might be considered the optimal ‘reference’.

The PBWmf + MBW curves (Fig. 2, Table 1) are entirely consistent with prevailing acute ventilation practice, in that they fully adhere to the male and female Devine formulae, and in fact extend them down to around 130 cm (they were traditionally considered valid above 5 ft/152 cm [15, 16]). Below 130 cm, the PBWmf + MBW curves merge to fit the population median data. The contribution of this model is primarily that of extending down to the smallest body sizes without disturbing current ventilation practice. However, the value of retaining sex-specific body weight predictions may be questioned when other sources of variation embodied in the final tidal volume calculation are considered (see later).

The PBWu + MBW curve (Fig. 3, Table 2) removes patient sex from the model in the interests of simplification. This comes at the expense of a +4.9% (female) or −4.6% (male) deviation from the established PBW formulae across the region where these are generally considered valid (above 5 ft/152 cm). Of the three unisex PBW models, this offers the least percent error relative to both PBW Male and PBW Female. Therefore, this curve may be useful for applications where close conformance to current practice is important but specifying sex is impractical or inconvenient. Yet it prompts the question: how much discrepancy in tidal volume due to deviating from the ‘proven’ PBW formulae might result in a clinically-relevant change in outcome? A precise answer to this is unknown, but we can put it in the context of other sources of error.

One source of error is the accuracy of estimating height. True height can vary throughout normal daily activity by up to 2% [33, 34], which might be considered a baseline accuracy. In the hospital setting, standard methods of estimating patient height include: asking the patient (not always possible), dedicated height measurement devices, measuring height/length in-situ using a tape, estimating height from a more convenient body part, and basic visual estimation. Visual estimation is common, leading to average PBW errors of 10% in one adult study, while the best-performing height estimation method (the Chumlea knee-heel approach) led to an average PBW error of 5.8% [35].

Other contributors to variation in eventual tidal volume also need to be taken into account. A common protective tidal volume recommendation is 6–8 mL/kg_PBW_, offering a discretionary variation of 25*–*33%. This scaling factor was found to be protective when compared to 10–15 mL/kg_PBW_, but it is less clear how protective such a scaling factor is compared to intermediate volumes (8–10 mL/kg_PBW_). Furthermore, the major trials tended to compare two clinical’approaches’, rather than just two different tidal volume factors [4]. More fundamentally, recent analysis suggests that tidal volume may be less critical than driving pressure within a heterogeneous lung, emphasizing the importance of all elements of the lung-protective ventilation bundle rather than just tidal volume [2].

As a worst-case error contribution, the use of actual body weight for volume setting is still widespread in adults. Use of actual body weight in average height adults can result errors of +30% relative to established PBW, and more than +35% for shorter statures [35]. Even in centers where lung-protective ventilation is accepted, adults of short stature may be less likely to receive volumes consistent with protective recommendations [10].

In the context of the above observations, sex-specific weight predictions may be of questionable value to ultimate tidal volume selection. A unisex simplification to existing formulae would be a variation of at most 7% predicted weight (very short female). More generally, systematic deviations of 5*–*10% from the established PBW formulae may ultimately have minimal impact on initial tidal volume settings when considered amongst all other sources of clinical variation, particularly the ml/kg discretion and height estimation. Such generalizations are not intended to diminish the importance of setting safe tidal volumes during initial ventilator configuration, but rather to probe the ‘evidence’ supporting the established PBW formulae, in the interests of simplicity. As eloquently stated by Linares-Perdomo et al. in their adult PBW standardization proposal [15], “While it is not possible to identify a “true” or “correct” PBW, it is possible to choose a reasonable PBW equation that will eliminate this source of unwarranted variation in clinical research and practice”.

The PBWuf + MBW curve (Fig. 4, Table 3) also provides simplification while retaining consistency with the ARDSNet framework. In this case the single curve adheres to *PBW Female* formula, providing better alignment with lean body weight, while its adoption for males would result in under-volume rather than over-volume compared to the status quo. For a male patient, the result would be at most 10% less volume than if the *PBW Male* formula was used (at 5 ft/152 cm), or 6% less volume at an average male height (a discrepancy fully compensated for in tidal volume by a <0.5 mL/kg increase). Note that both male and female adult patients would receive volumes larger than if derived from population median weight. The PBWuf + MBW model is proposed for consideration as a standardized unisex PBW formula. It is offered as a practical compromise between simplification & conservative interpretation of ARDSnet practice, while also better reflecting adult lean body weight than established PBW formulae.

The MBW curve (Fig. 5, Table 4) offers an easily calculated indication of median population data which – if viewed in isolation – make it seem ideally suited to tidal volume titration in lung-protective ventilation. Compared to using the *PBW Male* formulae, direct replacement with the MBW curve would result in at most a 20% *reduction* in initial tidal volume, or 10% reduction compared to *PBW Female*. This lower volume would be fully compensated by an upward adjustment of less than 1 mL/kg_PBW_. So MBW may also be considered for lung protective ventilation, if complete departure from the established Devine formulae was contemplated.

This analysis has a numerous limitations. It is emphasized that the PBW models presented here are specific to lung protective ventilation, and are not appropriate for pharmacology or assessment of healthy body weight. The focus here is on lean body weight, with up to 10% underestimation tolerated. Whereas in healthy body weight assessment, adopting a median weight for a given height has been judged inappropriate, and instead use of age- and sex-specific BMI is recommended [23, 36–38]. It may also be questioned if median weight of modern populations should be used as a surrogate for lean body weight, given that increasing obesity may affect median values, particularly later in development. The WHO data sampled culturally and ethnically diverse populations, while the CDC population included growing children surveyed over 2 decades ago. Most importantly, the population median weights within the adult range were substantially leaner than those predicted by the prevailing relationships used in protective ventilation (i.e. Devine’s formulae describing healthy mature adults [19]). This suggests that the median population reference may be a better representation of lean body weight than the established PBW relationship. Another limitation is that direct height-weight data were not available at all statures, so age-based data were used to synthesize weight-from-height over these ranges. The age-mapping applied is equivalent to that of the McLaren method, which has 2 main limitations identified [23, 37, 38]: (1) it does not recognize age-related variation, of importance to nutrition assessment but less relevant to lean body weight estimation, and (2) it cannot offer prediction above the tallest median height, which is resolved in the MBW model by linear extrapolation at taller heights. The notion of mapping 50^th^ centile height to 50^th^ centile weight is broadly accepted, in contrast to doing so further from the median (as inherent to the Moore estimation method) [37, 38]. It can be seen in Fig. 1 that the median weights derived from each of the 4 data sets are in good alignment, despite the mix of direct and indirect height-weight information. Four nutritional studies [23, 36–38] directly compared weight-for-stature and BMI-for-age, and found correlation moderately good around the 50^th^ centile. Two of these studies included small children, with one concluding the 2 approaches are similar below the age of 8 years [23], while the other found agreement to be poorer at age 4–5 years than at younger ages [36]. Any such weakness here is alleviated by the *WHO 45–110 cm* data set, which provides direct weight-from-height data extending to heights corresponding to a 4–5 year age. Finally, the clinical robustness of using predicted weight rather than actual weight in infants may be questioned: will any potential safety benefits be confounded by the particular challenge of measuring length in babies, or the complication of the prediction process? The analysis presented here does not touch on this question, but simply defers to the recent consensus recommendation [6] in favor of using predicted body weight in pediatric ventilation, and offers a means of doing so without reference to growth charts.

## Conclusions

Four alternate lean body-weight predictions from height have been presented, each of which spans the range from premature infant through to fully-grown adult. The piecewise linear relationships are simple to calculate, with 4 breakpoints across the entire height range. The intended application is to simplify and facilitate the adoption of lung-protective ventilation, including smaller body sizes. Error targets were chosen consistent with this application. The 4 alternate PBW models offer degrees of departure from the body weight prediction employed in the landmark ARDSNet trial. It is proposed that the PBWuf + MBW model, which adopts the Devine PBW Female formula as the adult unisex prediction, be considered for use in protective ventilation. This model acknowledges current practice and offers clinical simplification, while providing lean body weight estimation down to pre-term infants. If adherence to current practice is paramount, the PBWmf + MBW model simply adds a pediatric extension to the traditional PBW formulae. If complete departure from the established PBW formulae is acceptable, the MBW curve best reflects lean body weight for all body sizes and may therefore better estimate resting metabolic demand, particularly for tall patients for whom the established PBW formulae increasingly over-estimate lean body weight. The analysis presented is generalized & theoretical; further research is needed to assess any clinical utility of the proposed models.

## Additional file

Additional file 1: Predicted body weight calculator for the 4 models presented, and for the Devine 1974 male/female formulae. This spreadsheet includes the derived population median ‘reference’ curve, the 4 weight-height population data sets, calculators for the 4 presented models, and the Devine 1974 PBW Male/Female predictions for comparison. Also included are imperial conversions for the piece-wise linear formulae constants and coefficients. (XLSX 307 kb)

## Abbreviations

ARDS: Acute respiratory distress syndrome
ARDSNet: ARDS Network (details at http://www.ardsnet.org/)
CDC: US Centers for Disease Control and Prevention
C_rs_: Compliance of the respiratory system
IBW: Ideal body weight
ICU: Intensive care unit
MBW: Median Body Weight from population growth data
mL/kg_PBW_: millilitres/kilogram predicted body weight
MRI: Magnetic resonance imaging
PBW Male: PBW Female: Predicted Body Weight as used in ARDSNet study
PBWmf + MBW: Adult male/female Predicted Body Weight per Devine, transitioning to pediatric Median Body Weight
PBWu + MBW: Adult unisex Predicted Body Weight per Devine male–female average, transitioning to pediatric Median Body Weight
PBWuf + MBW: Adult unisex Predicted Body Weight per Devine ‘female’, transitioning to pediatric Median Body Weight
PEEP: Positive end-expiratory pressure
WHO: World Health Organization
